# Low Dose Vaccination with Attenuated *Francisella tularensis* Strain SchuS4 Mutants Protects against Tularemia Independent of the Route of Vaccination

**DOI:** 10.1371/journal.pone.0037752

**Published:** 2012-05-25

**Authors:** Dedeke Rockx-Brouwer, Audrey Chong, Tara D. Wehrly, Robert Child, Deborah D. Crane, Jean Celli, Catharine M. Bosio

**Affiliations:** 1 Tularemia Pathogenesis Section, Laboratory of Intracellular Parasites, Rocky Mountain Laboratories National Institute of Allergy and Infectious Diseases/National Institutes of Health, Hamilton, Montana, United States of America; 2 Immunity to Pulmonary Pathogens Section, Laboratory of Intracellular Parasites, Rocky Mountain Laboratories, National Institute of Allergy and Infectious Diseases/National Institutes of Health, Hamilton, Montana, United States of America; National Institute of Allergy and Infectious Diseases, United States of America

## Abstract

Tularemia, caused by the Gram-negative bacterium *Francisella tularensis*, is a severe, sometimes fatal disease. Interest in tularemia has increased over the last decade due to its history as a biological weapon. In particular, development of novel vaccines directed at protecting against pneumonic tularemia has been an important goal. Previous work has demonstrated that, when delivered at very high inoculums, administration of live, highly attenuated strains of virulent *F. tularensis* can protect against tularemia. However, lower vaccinating inoculums did not offer similar immunity. One concern of using live vaccines is that the host may develop mild tularemia in response to infection and use of high inoculums may contribute to this issue. Thus, generation of a live vaccine that can efficiently protect against tularemia when delivered in low numbers, e.g. <100 organisms, may address this concern. Herein we describe the ability of three defined, attenuated mutants of *F. tularensis* SchuS4, deleted for FTT0369c, FTT1676, or FTT0369c and FTT1676, respectively, to engender protective immunity against tularemia when delivered at concentrations of approximately 50 or fewer bacteria. Attenuated strains for use as vaccines were selected by their inability to efficiently replicate in macrophages *in vitro* and impaired replication and dissemination in vivo. Although all strains were defective for replication *in vitro* within macrophages, protective efficacy of each attenuated mutant was correlated with their ability to modestly replicate and disseminate in the host. Finally, we demonstrate the parenteral vaccination with these strains offered superior protection against pneumonic tularemia than intranasal vaccination. Together our data provides proof of principle that low dose attenuated vaccines may be a viable goal in development of novel vaccines directed against tularemia.

## Introduction

*Francisella tularensis* is a Gram-negative, facultative intracellular, bacterium, whose species includes subspecies *tularensis* (also known as Type A), subspecies *holarctica* (also known as Type B), and subspecies *mediasiatica*. While subspecies *tularensis* is considered highly virulent for humans [1], [2], subspecies *holarctica* can cause disease in humans that is not typically lethal [3], and subspecies *mediasiatica* is considered avirulent in humans [4]. Additionally, the *F. novicida* species is avirulent in the immunocompetent host [3] and *F. philomiragia* is considered avirulent in humans [4].

Following inoculation by a variety of routes, both Type B and Type A subspecies can cause the disease coined tularemia. There are several forms of tularemia, dependent on the route by which the host was exposed to organism. Among these, pneumonic tularemia (acquired following inhalation of the bacterium) mediated by Type A subspecies is widely considered the most dangerous. This is, in part, due to the small numbers of organisms required to cause disease, e.g. 15–20 bacteria [5]. Further, during early stages of infection, when administration of antibiotic is ideal, there are few pathological changes in the lung to signal disease [6]. Thus, diagnosis and execution of treatment may be delayed until the organism has disseminated and caused wide spread, potentially septic infection.

Although there are only approximately 100 cases of tularemia per year in the United States and the incidence worldwide is largely unknown, interest in tularemia has increased over the past 10 years (http://www.cdc.gov/tularemia/statistics/state.html). This interest stems from development and use of virulent *F. tularensis* as a biological weapon during the mid 20^th^ century [7]. In response to development and history of weaponized *F. tularensis*, there is renewed effort to develop novel vaccines that effectively protect against tularemia, especially the pneumonic form.

To date, the most successful vaccine generated for protection against tularemia was derived from a highly passaged strain of subspecies *holarctica* [8]. The Live Vaccine Strain (LVS) has been shown to protect against parenteral infection with *F. tularensis* subsp. *tularensis* in a wide variety of hosts, including humans [5], [8], [9]. Unfortunately, a number of drawbacks associated with the vaccine have precluded its licensure in the United States, among which is the unknown mechanism of attenuation in LVS. Thus, the risk of unexpected reversion to virulence is a possibility and difficult to control. Furthermore, LVS failed to uniformly protect against pneumonic tularemia in humans [10]. Finally, when delivered in high concentrations, LVS can cause mild tularemia and thus presents an undesirable sequela of vaccination [5], [10].

One possible explanation for the inability of LVS to adequately protect against infection with aerosolized Type A *F. tularensis* is that key antigens required for protection against *F. tularensis* may not be present in LVS. The failure of closely related pathogens to fail in engendering complete protection against more virulent species has been documented in the literature and could certainly hold true for the poor protection offered by LVS against pneumonic tularemia [11], [12]. The complete genome of both LVS and a representative strain of Type A, SchuS4, have been sequenced and annotated. Although the genomes are very similar, there are approximately 35 genes that encode different protein sequences between LVS and SchuS4 [13]. Since the function of many of these proteins is not defined, it is possible that they may represent important protective antigens. Given these differences and possibilities, there has been significant effort to develop defined, attenuated strains of SchuS4 as vaccines with the hope that they possess a more comprehensive array of protective antigens. However, given the virulence of SchuS4, one attractive attribute of vaccination with an attenuated strain would be that only small numbers of bacteria are required to mediate effective protection against challenge with wild type *F. tularensis*.

Previously, we described generation of two attenuated strains of SchuS4 which resulted from single, targeted mutation of defined genes, i.e. the FTT0369c and FTT1676 loci [14]. These genes encode for proteins of unknown function and were identified via transcriptional profiling of the bacterium during its intracellular lifecycle. Both strains were attenuated for replication in primary cells *in vitro* and their ability to cause lethal disease *in vivo*. The goal of the current study was to determine if either or both of these attenuated SchuS4 strains, in addition to a third mutant generated by deleting both the FTT0369c and FTT1676 genes, could act as vaccines against infection with wild type SchuS4. Here we demonstrate that low vaccinating inoculums of *F. tularensis* SchuS4ΔFTT0369c or *F. tularensis* SchuS4ΔFTT1676 efficiently protected against both intradermal and intranasal infection with wild type SchuS4. Combined deletion of the FTT0369c and FTT1676 genes did not improve vaccine efficacy. Thus, our data provide support for the generation of defined attenuated mutants of Type A *F. tularensis* that provide protection against infection with wild type SchuS4 without the requirement of high inoculums during vaccination.

## Materials and Methods

### Bacteria

*Francisella tularensis* subsp. *tularensis* strain SchuS4 was kindly provided by Dr. Rick Lyons (Colorado State University, Ft. Collins, CO). The in-frame single gene deletion mutants SchuS4ΔFTT0369c and SchuS4ΔFTT1676 have been described previously [14]. To generate a double ΔFTT0369cΔFTT1676 mutant of Schu S4, electrocompetent SchuS4ΔFTT1676 were prepared, electroporated with purified pJC84ΔFTT0369c plasmid [14] and plated on kanamycin-containing (10 µg/ml) modified Mueller-Hinton (MMH) plates as described [14], to select for plasmid integration. Kanamycin-resistant colonies were subjected to sucrose counter-selection as described [14], and sucrose-resistant clones were analyzed for loss of kanamycin resistance and allelic replacement within the correct chromosomal locus, using primers JC614 (5′-GCTTGAGGGTGCATTAG-3′) and JC615 (5′-GGTATCTCAGGAGGTGTG-3′) and primers JC610 (5′-GCGAGATCTGGCTCGCTACGCTGTGACTGCCAAG-3′) and JC613 (5′-GCGGTCGACGGTGTGTCTAGATGTGCTC-3′), respectively [14]. Independent clones carrying both in-frame deletions of the FTT1676 and FTT0369c loci were isolated and used for further studies.

Bacteria used for *in vivo* infection were propagated as previously described [15]. Briefly, all bacteria were cultured in modified Mueller-Hinton broth (Mueller-Hinton broth supplemented with CaCl_2_, MgCl_2_, 0.1% glucose, 0.025% ferric pyrophosphate and 2% Medium Enrichment [50% glucose, 167 mM L-cysteine-HCl, 68 mM L-glutamine, 3 mM adenine, 376 µM nicotinamide adenine dinucleotide, 7 µM Vitamin B_12_, 217 µM thiamine pyrophosphate, 160 µM guanine-HCl, 50 µM ferric nitrate, 95 µM aminobenzoic acid, 9 µM thiamine hydrocholride]) at 37°C with constant shaking overnight, aliquoted into 1 ml samples, frozen at −80°C and thawed just prior to use as previously described [16]. Frozen stocks were titered by enumerating viable bacteria from serial dilutions plated on MMH agar as previously described [17], [18]. The number of viable bacteria in frozen stock vials varied less than 1% over a 12 month period.

### Mice

Female C57Bl/6J and Balb/c mice 6–8 weeks of age were purchased from Jackson Laboratories (Bar Harbor, ME). Groups of 5–10 mice were used in each experiment as indicated. Mice were housed in BSL-2 and BSL-3 containment facilities at the Rocky Mountain Laboratories and provided with food and water *ad libitum*. All experiments with animals were conducted following approved protocols and under the guidance of the ACUC at Rocky Mountain Laboratories. Following infection with the indicated strains of SchuS4, all animals were regularly monitored for signs of illness. When signs of impending mortality were observed, animals were immediately euthanized.

### Generation and infection of bone marrow derived macrophages

Bone marrow derived macrophages (BMMs) were propagated from C57Bl/6J mice as previously described [14]. Bone marrow cells were isolated from femurs of 6–10 week-old, C57BL/6J female mice and differentiated into macrophages for 5 days at 37°C and 7% CO_2_, in 1 g/L glucose Dulbecco's Modified Eagle Medium (DMEM, Invitrogen) supplemented with 10% fetal bovine serum (FBS, Invitrogen), 10% L929-conditioned medium, and 2 mM L-glutamine (cDMEM) in non-tissue culture-treated Petri dishes. After 5 days, loosely adherent BMMs were washed with PBS, harvested by incubation in chilled cation-free PBS supplemented with 1 g/L D-glucose on ice for 10 min, resuspended in complete medium and replated in 24-well cell culture-treated plates at a density of 1×10^5^ macrophages/well. BMMs were further incubated at 37°C under 7% CO_2_ atmosphere for 48 h, replenishing with complete medium 24 h before infection.

For infections, bacteria grown on MMH agar plates for 3 days were resuspended in MMH broth, diluted in complete medium and 0.5 ml were added to chilled BMMs at an appropriate multiplicity of infection (MOI). Bacteria were centrifuged onto macrophages at 400× g for 10 min at 4°C, and infected BMMs incubated for 20 min at 37°C under 7% CO_2_ atmosphere including an initial, rapid warm-up in a 37°C water bath to synchronize bacterial uptake. Infected BMMs were then washed 5 times with DMEM to remove extracellular bacteria, incubated for 40 min in complete medium, and then for an additional 60 min in cDMEM containing 100 µg/ml gentamicin to kill extracellular bacteria. Thereafter infected BMMs were incubated in gentamicin-free medium until processing.

To quantify intracellular colony forming units (CFUs), BMMs (1×10^5^/well) were infected as described above, washed 3 times with sterile PBS then lysed with 1 ml of sterile deionized water for 3 min at room temperature, followed by repeated pipetting to complete lysis. Serial dilutions of the lysates were immediately plated onto MMH agar plates. Plates were incubated for 3 days at 37°C under 7% CO_2_ before enumeration of colony forming units (CFUs). The number of viable intracellular bacteria per well was determined in triplicate for each condition and at least 2 independent experiments were performed.

### Infection of mice

Balb/c mice were intranasally (10 CFU) or intradermally (50 CFU) infected with *F. tularensis* strain SchuS4, ΔFTT0369c, ΔFTT1676, or ΔFTT0369cΔFTT1676 as previously described [14], [15]. In additional experiments animals were challenged with the indicated doses of *F. tularensis* ΔFTT0369c SchuS4 intradermally. Briefly, mice were anesthetized with a single intraperitoneal injection of 12.5 mg/ml ketamine+3.8 mg/ml xylazine immediately prior to infection. For intranasal infections, bacteria were diluted in PBS to the indicated inocula and were administered in a total volume of 25 µl alternating between nares. For intradermal infection, bacteria were diluted in PBS to the indicated inocula and administered between the dermal sheets of the ear using a 30G needle and 300 cc syringe in a total volume of 10 µl. All inocula were plated on MMH agar and incubated at 37°C/7% CO_2_ for 48 hours to determine actual dose administered to the animal.

### Assessment of bacterial loads

Bacterial loads in target organs were determined as previously described [15]. Briefly, the indicated tissues were aseptically collected and placed in sterile PBS. Organs were immediately homogenized by grinding tissues through a sterile S/S Type 304 #60 wire mesh screen (Billeville Wire Cloth Co., Cedar Grove, New Jersey) using a 3 ml syringe plunger. Screens were rinsed with approximately 0.5 ml PBS and the resulting homogenate was immediately serially diluted in PBS. Dilutions of homogenate were plated on MMH agar and incubated at 37°C/7% CO_2_ for 48–72 hours and individual colonies were enumerated.

### Statistical Analysis

Significance in survival between groups was determined using Mantel-Cox (log-rank) analysis with significance set at p<0.05. Significant difference in bacterial numbers in tissues among groups of animals was determined using one way ANOVA followed by Bonferroni's post-test with significance set at p<0.05. LD50 was determined using the Spearman-Karber method [19].

## Results

### Infectivity and replication of Schu S4ΔFTT0369 and Schu S4ΔFTT1676 in vivo

**Figure 1:**
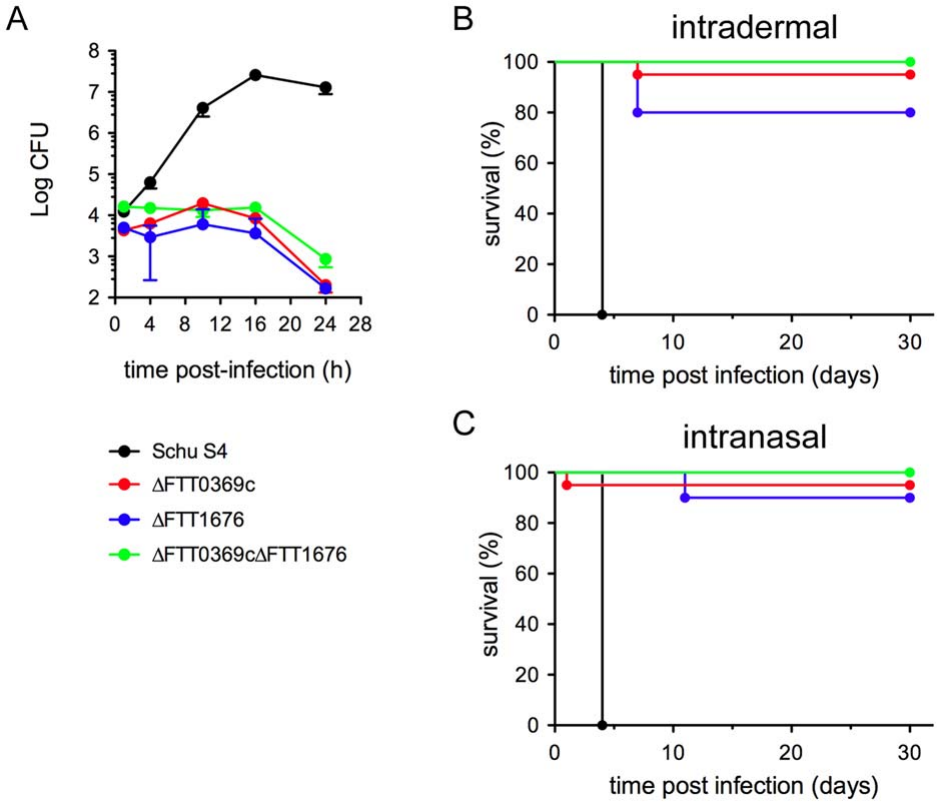
*In vitro* and *in vivo* attenuation of SchuS4ΔFTT0369c, ΔFTT1676 and ΔFTT0369cΔFTT1676 mutants. (**A**) Intracellular growth of *F. tularensis* SchuS4, ΔFTT0369c, ΔFTT1676 and ΔFTT0369cΔFTT1676 strains in murine BMMs derived from C57Bl/6J mice. BMMs were infected with either strains, as described in the Materials and Methods section, and intracellular viable bacteria were enumerated at the indicated times points after infection. Values are means ± SD of a representative experiment performed in triplicate. Data is representative of 2 experiments of similar design. (B–C) Balb/c mice (n = 5–10/group) were infected intradermally (B) or intranasally (C) with approximately 50 CFU or 10 CFU, respectively, of the indicated strains of *F. tularensis* SchuS4. Mice were regularly monitored up to 30 days after infection and euthanized at the first sign of irreversible illness.

Numerous studies have established the importance of intracellular proliferation in the virulence of various subspecies and strains of *Francisella tularensis* [14], [20]–[27]. Recently, we identified two genetic loci (FTT0369c and FTT1676) that were induced during the intracellular cycle of the virulent strain SchuS4 and required for intracellular proliferation [14]. Individual deletion of these loci in SchuS4 generated mutant strains that were attenuated in mice [14]. Given their attenuation *in vivo*, we sought to examine whether inoculation with these mutants resulted in productive infection or if the mutant strains were immediately eradicated at the site of infection. Mice were infected intradermally or intranasally with a target inoculum of 50 or 10 CFU, respectively. Consistent with our previous observations [14], 95% and 80% of mice infected intradermally with either ΔFTT0369c or ΔFTT1676 survived infection, respectively (Figure 1B). Similarly, 95% of animals receiving ΔFTT0369c intranasally and 90% of mice intranasally infected with ΔFTT1676 survived infection (Figure 1C and [14]).

**Figure 2:**
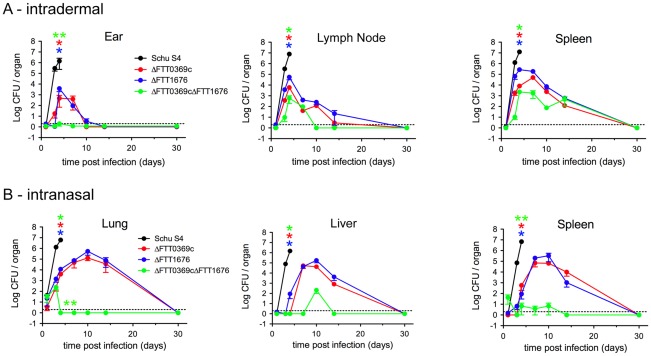
*In* vivo proliferation and dissemination of SchuS4ΔFTT0369c, ΔFTT1676 and ΔFTT0369cΔFTT1676 mutants following parenteral or pulmonary inoculations. Balb/c mice (n = 5–10/group) were infected intradermally (A) or intranasally (B) with approximately 50 CFU or 10 CFU, respectively, of the indicated strains of *F. tularensis* SchuS4. Mice were regularly monitored up to 30 days after infection and euthanized at the first sign of irreversible illness. At the indicated time points, organs were harvested and assessed for bacterial burdens as described in the materials and methods. Error bars represent SEM. Data is representative of two experiments of similar design. * indicates a p value<0.05 compared to SchuS4. ** indicates a p value<0.05 compared to all other groups. Asterisks colors refer to the strain tested for statistically significant difference.

We next assessed bacterial replication and dissemination from the site of infection to peripheral organs. Following intradermal infection, replication of both ΔFTT0369c and ΔFTT1676 was detected in the ear tissue. As expected, numbers of ΔFTT0369c and ΔFTT1676 retrieved from the ear were significantly less than those found in animals infected with wild type SchuS4 (Figure 2A). Similarly, we isolated significantly fewer numbers of ΔFTT0369c and ΔFTT1676 compared to wild type SchuS4 from both the draining lymph node and spleen of intradermally infected animals (Figure 2A). In the ear, ΔFTT0369c and ΔFTT1676 underwent replication up to day 5 or 7 of infection, respectively. Neither mutant was detected in the ear after day 10 of infection (Figure 2A). Clearance of ΔFTT0369c and ΔFTT1676 bacteria from peripheral organs was obvious after day 7 of infection (Figure 2A).

We also assessed replication and dissemination following intranasal infection. Similar to intradermal infection, significantly fewer bacteria were retrieved from the lungs of mice infected with either ΔFTT0369c or ΔFTT1676 compared to animals infected with wild type SchuS4 on day 3 and 4 of infection (Figure 2B). Dissemination of ΔFTT0369c and ΔFTT1676 to liver and spleen was delayed in comparison to wild type SchuS4. However, once bacteria arrived at these tissues they replicated to similar levels as those observed on day 3 of infection in animals infected with wild type SchuS4 (Figure 2B). Signs of control of replication of ΔFTT0369c and ΔFTT1676 were apparent by day 10 after infection in each tissue and all bacteria were cleared by day 30 (Figure 2B).

### F. tularensis ΔFTT0369c and ΔFTT1676 mutants protect against intranasal and intradermal SchuS4 infection

**Figure 3:**
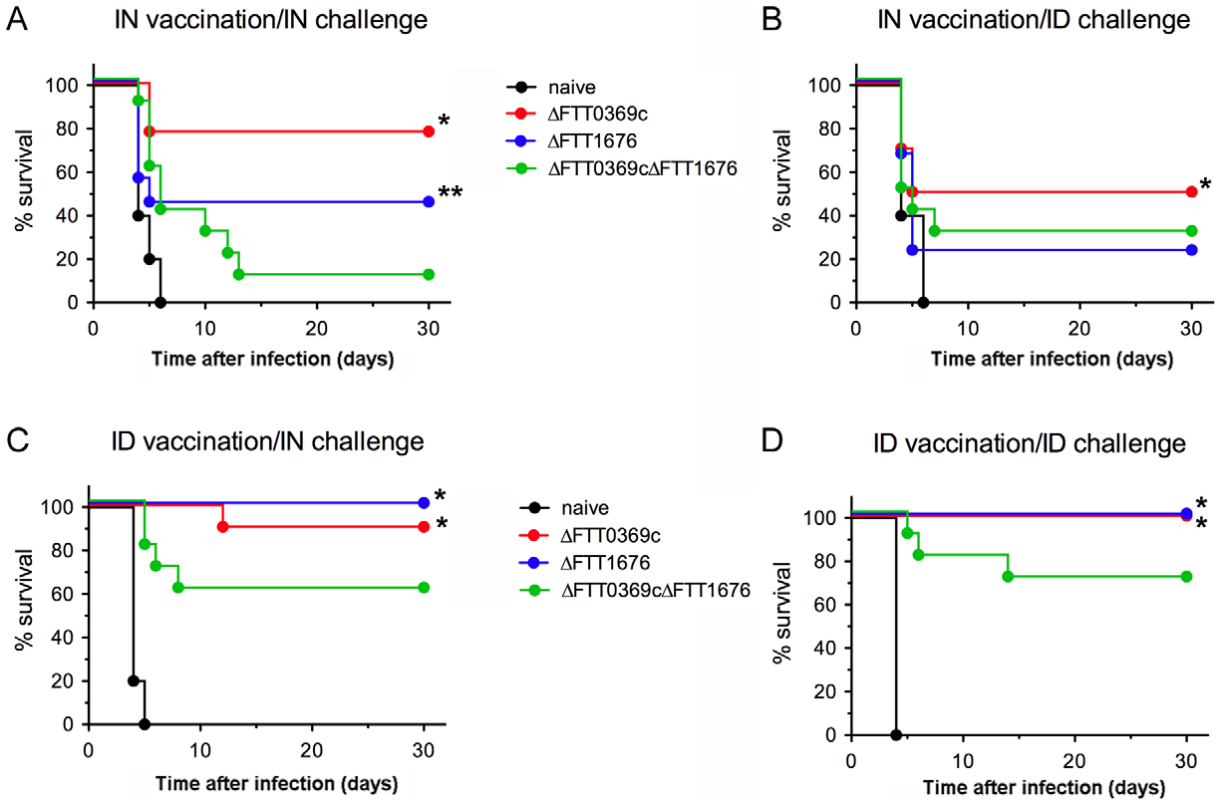
Protective capability of SchuS4ΔFTT0369c, ΔFTT1676 and ΔFTT0369cΔFTT1676 mutants against parenteral and pulmonary challenge with wild type SchuS4. Balb/c mice (n = 10/group) were infected intradermally or intranasally with approximately 50 CFU or 10 CFU, respectively, of the indicated strains of *F. tularensis* SchuS4. Forty-five days after infection, mice were challenged with approximately 50 CFU or 10 CFU of wild type *F. tularensis* SchuS4 intradermally or intranasally, respectively. Mice were regularly monitored up to 30 days after infection and euthanized at the first sign of irreversible illness. Data are representative of 2 experiments of similar design. * = p<0.05 compared to all other groups. ** = p<0.05 compared to naïve animals and mice vaccinated with ΔFTT0369cΔFTT1676.

We next determined if animals exposed to low doses of either ΔFTT0369c or ΔFTT1676 mutants that survived and cleared infection were protected against secondary challenge with wild type SchuS4. Previous studies have shown that intranasal immunization offers superior protection compared to parenteral routes, intradermal or subcutaneous [28], [29]. Thus, we first examined the ability of mice initially infected intranasally with attenuated SchuS4 strains to survive secondary intranasal or intradermal infection with wild type SchuS4. Mutant strain ΔFTT0369c offered the best protection against wild type SchuS4 infection. Specifically, significantly more animals previously exposed to ΔFTT0369c bacteria survived intranasal infection with SchuS4 compared to mice first challenged with ΔFTT1676 bacteria, 80% survival versus 50% survival, respectively (Figure 3A). Similarly, intranasal inoculation with ΔFTT0369c organisms resulted in significantly greater protection against intradermal challenge with SchuS4 compared to mice previously infected with ΔFTT1676. Specifically, 50% of animals vaccinated with ΔFTT0369c survived challenge with wild type SchuS4 compared to only 25% surviving animals vaccinated with ΔFTT1676 (Figure 3B). Initially, this suggested that challenge via the same route of vaccination may be required for optimal survival and that the ΔFTT0369c mutant engendered better protection against SchuS4 infection compared to the ΔFTT1676 strain.

We next assessed protection in mice that were first challenged with attenuated strains of SchuS4 intradermally. Regardless of the route of challenge with wild type SchuS4, more animals that were challenged intradermally with attenuated strains of SchuS4 survived infection compared to those that received attenuated SchuS4 intranasally (Figure 3). Specifically, significantly greater numbers of animals, i.e. 100%, exposed to either ΔFTT0369c or ΔFTT1676 bacteria intradermally, were protected against wild type SchuS4 infection when delivered via the same route compared to naïve controls (Figure 3D). Further, significantly more mice inoculated intradermally with ΔFTT0369c or ΔFTT1676 bacteria were protected from an intranasal challenge with wild type SchuS4 compared to naïve controls, i.e. 90% and 100%, respectively (Figure 3C). Thus, low dose intradermal vaccination with either attenuated strains of SchuS4 efficiently protected mice against both intranasal and intradermal infection with wild type SchuS4.

### Effect of the combination of the ΔFTT0369c and ΔFTT1676 mutations on in vitro infection

Although both mutant strains offered protection against infection with wild type SchuS4, at least one mutant (ΔFTT1676) caused a lethal infection in 20% of inoculated mice (Figure 1B). Thus, we hypothesized that a strain of SchuS4 deleted for both FTT0369c and FTT1676 genes may be more attenuated *in vivo*, but would still engender protection against infection with wild type SchuS4. To this end, we generated a double deletion mutant of both FTT0369c and FTT1676 loci and evaluated its ability to infect macrophages and replicate intracellularly *in vitro*. Compared to the parental SchuS4 strain, which grew by 3 orders of magnitude within BMMs over a 24 h time frame, intracellular viable numbers of the double mutant SchuS4ΔFTT0369cΔFTT1676 did not significantly increase during the first 16 h pi, and even decreased afterwards, similar to both single deletion mutants (Figure 1A). The double mutant did not show a stronger intracellular defect than the single deletion mutants, indicating that inactivation of these two genes does not have an additive effect within macrophages (Figure 1A).

### Virulence, replication and dissemination of the SchuS4ΔFTT0369cΔFTT1676 in vivo

We next assessed whether the ΔFTT0369cΔFTT1676 mutant was attenuated *in vivo*. All mice survived low dose infection with ΔFTT0369cΔFTT1676 regardless of the route of inoculation (Figure 1B–C). Given this apparent attenuation, we next determined if the ΔFTT0369cΔFTT1676 mutant underwent replication and/or dissemination following intradermal and intranasal infection.

In contrast to ΔFTT0369c and ΔFTT1676 mutants, we were unable to retrieve ΔFTT0369c ΔFTT1676 bacteria from the ears of intradermally infected animals (Figure 2A). However, assessment of the draining lymph node and spleen revealed dissemination and replication of the double mutant in each peripheral compartment. However, numbers of ΔFTT0369cΔFTT1676 bacteria were significantly lower than either single mutant at day 3 and day 7 in the lymph node and spleen, respectively (Figure 2A). Together this suggested that, although ΔFTT0369cΔFTT1676 bacteria were less competent for replication following intradermal infection compared to ΔFTT0369c or ΔFTT1676 strains, the double mutant was not completely defective for replication *in vivo*. Clearance of ΔFTT0369cΔFTT1676 bacteria in the draining lymph node occurred earlier than either single mutant with no bacteria observed in this tissue by day 10 of infection. However, control and clearance of ΔFTT0369cΔFTT1676 bacteria in the spleen was similar to that observed for infection mediated by either single mutant (Figure 2A).

In contrast to our inability to detect replication of the ΔFTT0369cΔFTT1676 mutant at the site of infection following intradermal infection, it was capable of limited replication in the lung during the first three days of infection (Figure 2B). However, ΔFTT0369cΔFTT1676 were readily controlled thereafter and we did not detect bacteria in the lung after this time point (Figure 2B). We also observed transient and modest dissemination and/or replication of the ΔFTT0369cΔFTT1676 mutant to the liver and spleen following intranasal infection with significantly fewer numbers retrieved from these organs at each time point tested compared to both ΔFTT0369c and ΔFTT1676 bacteria. Together these data suggest that both ΔFTT0369c and ΔFTT1676 are modestly attenuated *in vivo*, whereas the double mutant ΔFTT0369cΔFTT1676 is significantly impaired for replication and dissemination compared to single mutant strains.

### The SchuS4ΔFTT0369cΔFTT1676 mutant engenders minimal protection against intranasal and intradermal SchuS4 infection

We next determined if animals exposed to low doses of ΔFTT0369cΔFTT1676 bacteria were protected against secondary challenge with wild type SchuS4. Unlike animals inoculated with single mutants, protection engendered by ΔFTT0369cΔFTT1676 bacteria was very poor and significantly lower than animals vaccinated with either SchuS4 single deletion mutant. Specifically, only 10% of mice first challenged intranasally survived intranasal infection with wild type SchuS4 (Figure 3A). Similarly, significantly fewer mice (i.e. 30%) that were previously infected with ΔFTT0369cΔFTT1676 survived intradermal SchuS4 infection (Figure 3B). Data with the single mutants suggested that intradermal inoculation may provide superior protection against wild type SchuS4 challenge. Thus, we next assessed protection against SchuS4 challenge in mice previously inoculated with ΔFTT0369cΔFTT1676 bacteria intradermally. Similar to our observation that previous intradermal infection provided greater protection against SchuS4 challenge, 70% of animals receiving the ΔFTT0369cΔFTT1676 mutant survived secondary intradermal infection with SchuS4 and 60% survived intranasal infection with the wild type strain. However, despite this improved protection compared to intranasal inoculation, the double mutant was not as effective as either single mutant for protecting against wild type SchuS4 (Figure 3).

### LD50 and protective efficacy of F. tularensisΔFTT0369c in vivo

**Figure 4:**
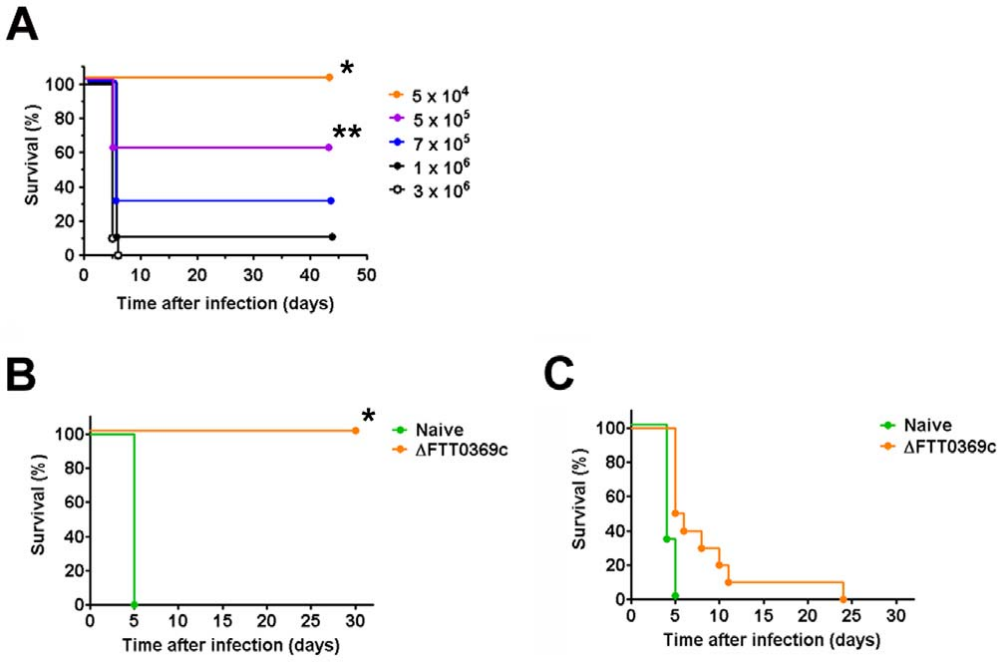
LD50 of SchuS4ΔFTT0369c and protective efficacy following increased challenge dose of wild type SchuS4. (A) Balb/c mice (n = 10/group) were infected intradermally with the indicated number of *F. tularensis* ΔFTT0369c. Mice were regularly monitored for up to 45 days after infection and euthanized at the first signs of irreversible illness. (B and C) Mice (n = 10/group) were challenged with approximately 5×10^4^ CFU intradermally. Forty-five days after infection mice were challenged intranasally with approximately 50 CFU (B) or 200 CFU (C) wild type SchuS4. Mice were regularly monitored for up to 30 days after infection and euthanized at the first signs of irreversible illness. * = p<0.05 compared to all other groups. ** = p<0.05 compared to mice receiving 7×10^5^, 1×10^6^ and 3×10^6^.

Although the ability of low doses of attenuated SchuS4 mutants to protect against virulent challenge is an important observation, there is a possibility that a modest increase in inoculating dose would result in fulminant, lethal disease. Obviously, this would be an undesirable feature for any vaccine. Regardless of route, inoculation of mice with 50 CFU of the ΔFTT0369c mutant did not result in any deaths. Furthermore, this strain offered the best protection against challenge with wild type SchuS4 following intranasal vaccination and similar protection following intradermal vaccination compared to the other single deletion mutant ΔFTT1676. This suggested that ΔFTT0369c may represent a viable vaccine strain for protection against wild type SchuS4. Thus, we next determined the LD50 of ΔFTT0369c and protection engendered by this strain when delivered at doses higher than 50 CFU. Significantly greater numbers of mice challenged with approximately 5×10^4^ CFU ΔFTT0369c survived, i.e. 100%, compared to all other doses tested (Figure 4A). Similarly, challenge with approximately 5×10^5^ CFU ΔFTT0369c resulted in 60% survival and significantly more living mice compared to animals challenged with 1–3×10^6^ CFU ΔFTT0369c (Figure 4A). Using the Karber method we calculated that that LD50 of intradermal infection with ΔFTT0369c was approximately 6.3×10^5^ CFU.

We next determined if high dose vaccination with Δ FTT0369c was capable of protecting animals against intranasal infection with SchuS4. Similarly to results observed in mice that received 50 CFU ΔFTT0369c intradermally, 100% of animals vaccinated intradermally with 5×10^4^ ΔFTT0369c survived intranasal infection with 50 CFU wild type SchuS4 (Figure 4B). However, intradermal vaccination with 5×10^4^ CFU Δ FTT0369c failed to protect animals against exposure to higher numbers (200 CFU) of wild type SchuS4 (Figure 4C).

## Discussion

The study presented herein demonstrated that inoculation of low doses of defined, attenuated mutants of *F. tularensis* strain SchuS4 could protect against parenteral and intranasal challenge with fully virulent wild type SchuS4. Furthermore, we confirmed that inoculation of higher doses of one attenuated mutant, i.e. up to 3log_10_ higher, did not result in adverse events in the host and offered a similar level of protection against intranasal challenge of up to 50 CFU wild type SchuS4. Thus, our data suggest that use of a defined, attenuated SchuS4 mutant may not require inoculation of extremely high numbers of the vaccinating strain. Rather, effective protection can be achieved with relatively low doses of attenuated organisms. Furthermore, our data demonstrate that increase in vaccinating inoculum does not correlate to increased protection against wild type SchuS4 and suggests that generation of protective immunity against tularemia requires features that are independent of “antigen load”.

There are a number of strategies for the generation of novel vaccines directed against tularemia. Although LVS can offer some protection against challenge with fully virulent *F. tularensis*, one detraction of LVS for current use is the ill-defined nature of its attenuation. Paired with a spontaneous ability to undergo a phase shift which negatively affects its protective efficacy, LVS is no longer a licensed vaccine for use against tularemia in the United States [9]. In response to these undesirable attributes of LVS, new efforts have been placed on developing defined, attenuated mutants of homologous strain, i.e. *F. tularensis* subsp. *tularensis*, which effectively protect against tularemia.

Several laboratories have reported success in generating defined Type A *F. tularensis* mutants that can protect against challenge with virulent *F. tularensis*. However, vaccination with these attenuated strains was typically only successful against intradermal infection with Type A *F. tularensis* with minimal to no protection against aerosol/intranasal infection. For example, *F. tularensis* deletion mutant ΔFTT0918 efficiently protected 100% of animals against intradermal infection with wild type *F. tularensis*, but only around 30% of mice vaccinated with this strain survived aerosol infection [30]. Similarly, a purine auxotroph mutant (*F. tularensis* Δ*purMCD*) successfully protected all mice from secondary parenteral infection with wild type SchuS4, but failed to protect more than 14% of animals against pneumonic tularemia [31].

There are two examples in which immunization with attenuated Type A *F. tularensis* offered greater protection against challenge with wild type strains compared to earlier reports. First, vaccination with bacteria deleted of the gene encoding a hypothetical lipoprotein (FTT1103) successfully protected against intranasal challenge of wild type *F. tularensis* SchuS4 [32]. A later report demonstrated that a single intradermal immunization with *F. tularensis* ΔFTT0918Δ*clpB* could protect approximately 40% of Balb/c mice from aerosol infection with fully virulent SchuS4 [33]. Similarly, in the report presented herein all three mutants offered anywhere from 20–100% protection against intranasal or intradermal challenge with 10–50 SchuS4 organisms, respectively (Figure 3). However, it is important to note that successful protection against SchuS4 utilizing the mutants discussed in the current report was achieved with a single dose of approximately 50 CFU of the vaccinating strain. Furthermore, we also established that animals inoculated with up to 5×10^4^ CFU of SchuS4ΔFTT0369c did not succumb to disease following infection and were readily protected against a low dose intranasal challenge with wild type SchuS4. This suggests that a fairly wide range of vaccinating doses may be used to establish equivalent protection. In contrast, others have had to use anywhere from 10^5^–10^8^ CFU of attenuated SchuS4 strains to achieve similar protection [30].

Interestingly, unlike other studies, vaccination via the same route as infection with wild type SchuS4 was not required to generate protective immunity. Rather, intradermal vaccination with any of the three mutants elicited a superior protection against both intradermal and intranasal challenge with SchuS4 compared to animals that received attenuated strains via the intranasal route (Figure 3). Thus, our data suggests that intranasal vaccination is not required for protection against pneumonic tularemia. This is in agreement with our and others earlier investigation with LVS and attenuated *F. tularensis* mutants in which effective protection following intranasal infection with SchuS4 was observed in mice vaccinated subcutaneously [34], [35]. Additionally, our data also suggests that intranasal immunization with live bacteria did not promote similar immune responses as those generated following intradermal vaccination. This idea is in line with previous reports from Woolard *et al* in which intranasal immunization with LVS provoked fewer protective IFN-γ producing T cells compared to subcutaneous immunization [36].

Among the SchuS4 mutants tested in this study, we observed differences in their ability to engender protective immunity. Regardless of the route of vaccination the single mutants offered significantly better protection against SchuS4 infection compared to the ΔFTT0369cΔFTT1676 double mutant. Early studies suggested that viable, replicating organisms are required for elicitation of protective immunity against *F. tularensis*, presumably to allow full unveiling of protective antigens. Absence of bacterial replication might explain lack of development of effective memory immunity in studies in which killed bacteria were used as the vaccinating agent [37]. Thus, one could speculate that the differences in protection mediated by the attenuated strains of SchuS4 used in this study could have been attributed to differential infection and replication *in vitro*. However, all three mutants exhibited similarly impaired intracellular replication within macrophages *in vitro* (Figure 1A). By contrast, we found striking differences in the ability of these mutants to establish infection *in vivo*. While both ΔFTT0369c and ΔFTT1676 bacteria exhibited similar replication at the site of infection (lung or ear) and dissemination to and replication in peripheral organs (Figure 2), the ΔFTT0369cΔFTT1676 mutant underwent weak replication at the site of infection and minimal dissemination to peripheral organs (Figure 2). Thus, the failure of the ΔFTT0369ΔFTT1676 mutant to engender protection against wild type SchuS4 infection similar to that observed using single mutants can be correlated with its inability to replicate at the site of infection and/or peripheral organs. Furthermore, we demonstrate that vaccination with 5×10^4^ ΔFTT0369c resulted in similar numbers of mice capable of surviving challenge with 50 CFU wild type SchuS4 compared to those that were vaccinated with only 50 CFU of the ΔFTT0369c strain. However, animals vaccinated with 5×10^4^ ΔFTT0369c were not protected against infection with 200 CFU wild type SchuS4. Thus, our data also demonstrate that increase in “antigen” during vaccination does not correlate to increased protection. Rather, we suggest that specific, important, elements of protective immunity are not elicited by the ΔFTT0369c mutant and that this is independent of the vaccinating dose. Alternatively, we have recently shown that animals which have survived infection with wild type SchuS4 are not fully protected against secondary challenge with the same strain [38]. This suggests that wild type SchuS4 may possess an inherent ability to inhibit development of effective adaptive immunity and that this feature has not been altered in the single deletion mutants.

Importantly, our data also suggests that replication deficiency in macrophages *in vitro* does not necessarily correlate with a lack of infectivity *in vivo*, since both single mutants were capable of limited proliferation at the site of infection and dissemination despite being deficient for replication in macrophages. This is in agreement with previous reports examining defined deletion mutants of SchuS4 for infection of cells and virulence *in vitro* and *in vivo* [39], [40] and indicates that i) replication of *Francisella* within macrophages is only a component of the bacterium's ability to proliferate within host tissues and disseminate to peripheral organs and ii) other cell types or extracellular compartments likely support bacterial proliferation.

When comparing protective efficacy of the single mutants used in this study there was little difference between the strains when immunization occurred via the intradermal route. However, following intranasal vaccination, significantly more animals that were first exposed to ΔFTT0369c bacteria survived intranasal challenge with wild type organism compared to mice immunized with the ΔFTT1676 strain. Since both bacteria had similar patterns of replication and dissemination *in vivo*, these results may suggest that the way in which the mutants interacted with the immune system was different. Previously, it has been shown that wild type strains of *F. tularensis* potently suppress both inflammation and developing T cell responses. For example, we and others have observed that SchuS4 readily inhibits recruitment of inflammatory cells and production of cytokines associated with protective T cell response, e.g. IL-12 [16], [39], [41], [42]. Furthermore, it has also been reported that *Francisella* infection can result in degradation of both Major Histocompatibility Complex II (MHCII) and CD86 on the surface of antigen presenting cells [43]. These receptors are critical for antigen presentation and subsequent protective T cell responses directed against *Francisella*. Therefore, it is possible that the increased protective efficacy observed in mice immunized with ΔFTT0369c bacteria was partly due to the inability of this mutant to effectively inhibit inflammatory and/or T cell responses. In support of this hypothesis, we have recently shown that ΔFTT0369c induced secretion of IL-12p40 from primary dendritic cells, whereas infection with wild type SchuS4 failed to do so [41]. Further studies on the functions of the proteins encoded by the FTT0369c and FTT1676 loci may reveal molecular basis for the different protective efficacy of these mutants.

Together our data provide proof of principle that delivery of low doses of live, attenuated vaccine derived from fully virulent Type A *F. tularensis* subspecies can protect against challenge with virulent homologous strains. Furthermore, our results support previous work suggesting screening of mutants in multiple cell types for competence in replication may be necessary for predicting virulence and/or protective efficacy *in vivo*. Finally, these defined attenuated mutants of SchuS4 may also be useful in defining the role of specific host molecule, e.g. IL-12, or pathways that are required for survival of tularemia.
